# British Pharmaceutical Conference

**Published:** 1908-09-19

**Authors:** 


### Therapeutics.

### BRITISH PHARMACEUTICAL CONFERENCE.

The forty-fifth annual meeting of the British Pharmaceutical Conference, which was held at Aberdeen on September 15 and 16, was attended by a large gathering of pharmacists from all parts of the country, and the high character of the papers contributed is evidence that the progress of scientific pharmacy has not been retarded by the trade elements which of late years have been introduced as a result of competition from limited companies and co-operated societies. Mr. Robert Wright, in his presidential address, dealt mainly with the various difficulties with which chemists have to contend, and his comments on the traffic in quack remedies no doubt reflect the views of better-class pharmacists concerning this kind of trade. He advised every pharmacist to set himself steadfastly against quackery and refuse to countenance it in any shape or form. Statistics show that the annual value of stamped medicines sold is increasing at an enormous rate. The amount expended in remedies of this description in the years below mentioned was approximately as follows:—1860, £350,000; 1870, £580,000; 1880, £1,080,000; 1890, £1,740,000; 1900, £2,310,000; 1907, £2,620,000. Reckoning the population of the British Isles at about twenty-three millions in 1860, and thirty-nine millions in 1906, it follows that the amount spent in patent medicines per head is now about five times as great as it was half a century ago. After making full allowances for the increased spending power of the masses, these figures prove conclusively that notwithstanding the wide diffusion of knowledge, the spread of education, and the raising of the standard of intelligence among the people, the appeal of the quack and the charlatan to the credulity of the public meets with a readier response than ever. Mr. Wright characterised the entire system as nothing more or less than a most monstrous imposture from beginning to end, and believed that if it were possible to set on foot an impartial inquiry into the mischief wrought by this class of remedies it was certain that some startling facts would be brought to light. The remedy, in his opinion, rests with Parliament, and the production and sale of such medicine should be submitted to much severer restrictions than is the case at present. The outlets by means of which they find their way to the public should be limited by raising very considerably the cost of the patent-medicine license, and he suggested that a Board of Control, consisting largely of recognised authorities in the different branches of medicine, should be constituted, to which it should be made compulsory for the formula of every secret remedy to be submitted for registration before permission were given for its public advertisement.

Among the twenty-two papers contributed were several which contained important suggestions with regard to future editions of the British Pharmacopœia, and a brief reference to a few of these will be useful. In a paper dealing with standards for the alkaloidal drugs, J. C. Umney and C. T. Bennet, while appreciating the desirability of fixing standards of drugs as high as possible, insisted that this must be done with due regard to natural variations in them from season to season. In order to show the natural variation of alkaloidal strength in crude drugs and in that way to indicate, as far as possible, what limits are suitable, taking one season with another, the authors set out the percentage of alkaloids in different samples of the drugs examined over a period of three years. The variations are very considerable, and in some cases of great importance. Thus a few seasons ago practically no belladonna root was obtainable in commerce containing more than 0.3 per cent. of alkaloids, while at the present time the roots available in commerce contain as much as .55 per cent. The variation in coca is also enormous, but a normal average may be taken as being 0.5 per cent. Cinchona barks also vary greatly. Ipecacuanha is fairly uniform and generally yields from 2.0 to 2.5 per cent. of alkaloid soluble in chloroform. Dealing with liquid extracts, the authors expressed the opinion that it is extremely doubtful whether liquid extract of aconite will be included in the new British Pharmacopœia.

The electrolytic administration of drugs was dealt with in a short note by T. M. Clague. It is well-known that with decomposition of chemical compounds by passage of a current of electricity through their solutions the basic portion of the salt goes to the negative pole and the acid portion alone to the positive. He described how this fact had been utilised as a means of putting a drug just where it was wanted. Thus it has frequently been observed that small doses of magnesium sulphate cause the disappearance of warts, and this led Dr. Lewis Jones to try treating warts electrolytically with magnesium salts. The results were excellent. The entire absence of scar makes the method a much better one than the knife or caustic appliances, and if the warts are numerous several may be treated at once. Corns readily yield to negative current and sodium salicylate.

Formic acid and some of its salts was the subject of a paper by G. Leman, who discussed the comparative purity and mode of exhibition of these substances. As the result of his investigations the author came to the conclusion that on the whole the formic acid of commerce can be relied on as being reasonably free from impurities. He gave the following as the correct maximum doses:—Calcium formate 5 grs., lithium formate 5 grs., potassium formate 10 grs., sodium formate 10 grs.

A mode of preparation of a soluble ferric arsenate was suggested by F. B. Power and H. Rogerson. It is generally admitted that the iron arsenate of the pharmacopœias is a very unsatisfactory preparation for medicinal use, for it is variable in composition and has the disadvantage of being quite insoluble in water. The idea suggested itself to the authors that a soluble ferric arsenate would present decided advantages over the insoluble compound, and some experiments were therefore made for the production of such a soluble salt by the combination in suitable proportions of ferric citrate and sodium arsenate.

